# Polyisoprenylated Methylated Protein Methyl Esterase Is Both Sensitive to Curcumin and Overexpressed in Colorectal Cancer: Implications for Chemoprevention and Treatment

**DOI:** 10.1155/2013/416534

**Published:** 2013-07-01

**Authors:** Felix Amissah, Randolph Duverna, Byron J. Aguilar, Rosemary A. Poku, Nazarius S. Lamango

**Affiliations:** College of Pharmacy and Pharmaceutical Sciences, Florida A&M University, Tallahassee, FL 32307, USA

## Abstract

Inhibition of PMPMEase, a key enzyme in the polyisoprenylation pathway, induces cancer cell death. In this study, purified PMPMEase was inhibited by the chemopreventive agent, curcumin, with a *K*_*i*_ of 0.3 ***μ***M (IC_50_ = 12.4 ***μ***M). Preincubation of PMPMEase with 1 mM curcumin followed by gel-filtration chromatography resulted in recovery of the enzyme activity, indicative of reversible inhibition. Kinetics analysis with N-para-nitrobenzoyl-S-*trans,trans*-farnesylcysteine methyl ester substrate yielded *K*_*M*_ values of 23.6 ± 2.7 and 85.3 ± 15.3 ***μ***M in the absence or presence of 20 ***μ***M curcumin, respectively. Treatment of colorectal cancer (Caco2) cells with curcumin resulted in concentration-dependent cell death with an EC_50_ of 22.0 ***μ***g/mL. PMPMEase activity in the curcumin-treated cell lysate followed a similar concentration-dependent profile with IC_50_ of 22.6 ***μ***g/mL. In colorectal cancer tissue microarray studies, PMPMEase immunoreactivity was significantly higher in 88.6% of cases compared to normal colon tissues (*P* < 0.0001). The mean scores ± SEM were 91.7 ± 11.4 (normal), 75.0 ± 14.4 (normal adjacent), 294.8 ± 7.8 (adenocarcinoma), and 310.0 ± 22.6 (mucinous adenocarcinoma), respectively. PMPMEase overexpression in colorectal cancer and cancer cell death stemming from its inhibition is an indication of its possible role in cancer progression and a target for chemopreventive agents.

## 1. Introduction

Colorectal cancer is the third most commonly diagnosed cancer and the third leading cause of cancer-related deaths, accounting for about 610,000 deaths per year worldwide [1, 2]. Siegel and coworkers [1] projected a total of 143,460 new cases of colorectal cancer and 51,690 related mortalities in the US in 2012 [1]. Colon cancer development is a multistep process initiated by molecular alterations such as mutations in adenomatous polyposis coli (APC), K-ras, and/or p53 genes [3]. The tissue is then predisposed to subsequent transformation mainly through abnormal cell proliferation, angiogenesis, reduced apoptosis, and changes in growth factor activity [3]. Despite recent medical advances, colorectal cancer recurs in up to 50% of patients [4–6]. The prognosis for the advanced colorectal cancer is very poor due to liver metastasis [7, 8] as well as resistance to chemotherapy [9]. The metastasis has recently been shown to involve the activation of the Rho family of polyisoprenylated small GTPases [10]. These include RhoA and Rac1 which regulate actin cytoskeleton and cell migration [11]. RhoA stimulates the actin stress fiber formation and cell-cell adhesion, while Rac1 induces lamellipodia formation [10]. Enzymes of the polyisoprenylation pathway, which modify these proteins, have thus been the targets for anticancer drug development. Polyisoprenyl transferase inhibitors have been a major part of these efforts [12]. Similar efforts have explored the role of inhibiting polyisoprenylated protein methyl transferase (PPMTase) to curb cancer cell growth [13]. Polyisoprenylated methylated protein methyl esterase (PMPMEase, EC 3.1.1.1) hydrolyzes the ester products of PPMTase, thus counteracting the effects of PPMTase at the terminal only reversible reaction of the pathway [14]. PPMTase and PMPMEase thus appear to be pivotal regulating polyisoprenylated protein function. 

 Several food components such as flavonoids, phenolics, and polyphenols are chemopreventive [15] and are being used as dietary supplements to prevent colon cancer [16]. Use of these compounds at nontoxic doses inhibit, reduce, or delay carcinogenesis at its early stages [3]. One such compound is curcumin, the main bioactive constituent of turmeric spice derived from the rhizome of *Curcuma longa* (Zingiberaceae) [17]. Curcumin is a compound with anticancer [18, 19], anti-inflammatory [20], and antioxidant properties [21]. In rodent models, the compound inhibits the development of cancers of the skin, duodenum, tongue, colon, mammary, and prostate glands [22, 23]. Curcumin has also been reported to inhibit cell proliferation as well as inducing apoptosis in cancer cells [23, 24]. The anticancer potential of curcumin is limited by its poor bioavailability [25]. However, when ingested orally, a concentration as low as 0.2% can prevent the development of colon cancer [26]. The chemopreventive and antitumor effect of curcumin in colon cancer has been extensively studied and has been linked to the inhibition of cyclooxygenase-2 [27], aminopeptidase N [28], and antiangiogenesis [29]. Recent studies have revealed that curcumin inhibits human colon cancer cell growth by suppressing EGFR gene expression [30] as well as the Ras signaling pathway [31]. The effects on Ras signaling are interesting given that K-Ras gene mutations are implicated in about 50% of colon cancers cases [32]. Since Ras and other monomeric G-proteins are processed through the polyisoprenylation pathway in order to be fully functional, it is possible that compounds that interfere with the secondary modifications may have effects on carcinogenesis. Studies from our laboratory have established that PMPMEase inhibition induces cancer cell death [33, 34]. Given that aberrant activities of polyisoprenylated proteins play an important role in a majority of colon cancer progression cases [32] and PMPMEase inhibition has such a profound negative effect on cancer cell viability [33–35], the current study was aimed at determining if PMPMEase may constitute a pharmacological target for bioactive anticancer agents such as curcumin. This was determined by investigating PMPMEase susceptibility to curcumin inhibition and expression in colorectal cancer. Here, we report that PMPMEase is both inhibited by curcumin and is overexpressed in colorectal cancer implying that the chemopreventive effects of curcumin may be due at least in part to PMPMEase inhibition. 

## 2. Materials and Methods

### 2.1. Materials

Human colorectal adenocarcinomas (Caco-2) cells obtained from the American Type Culture Collection (Manassas, VA, USA) were cultured in Dulbecco's Minimum Essential Medium (Invitrogen, CA, USA), supplemented with 20% heat-inactivated fetal bovine serum, 15 mM HEPES buffer, 100 U/mL penicillin and 100 *μ*g/mL streptomycin, and 1% nonessential amino acids obtained from Invitrogen (Carlsbad, CA, USA). The cultures were incubated at 37°C in 5% CO_2_/95% humidified air. Curcumin (97% purity) was purchased from Merck (Whitehouse Station, NJ, USA).

### 2.2. Enzyme Assays

PMPMEase used for the assays was the same as that previously described [36, 37]. The substrate (RD-PNB) and curcumin were dissolved in DMSO. The enzyme assays and analysis were conducted as previously described [36, 37] except with a 15 min preincubation of the assay mixture with curcumin before the addition of substrate. RD-PNB (1 mM) was incubated at 37°C with the enzyme in the presence of curcumin in 100 mM Tris-HCl, pH 7.4 in a total incubation volume of 100 *μ*L. Reactions were stopped by adding 200 *μ*L of methanol and placing them on ice for at least 5 min before centrifugation at 5000 ×g for 5 min. The supernatant was analyzed by RP-HPLC. The product was separated from the substrate on a Hamilton PRP-1 RP-HPLC column (5 *μ*m particles, 4.1 mm ID × 50 mm) with UV detection at 260 nm. The mobile phase consisted of a linear gradient of acetonitrile in 0.1% ethanolamine, from 30% at the start of the separation to 95% in 1 min. This was then maintained for a further 2 min before a 0.5 min reequilibration at 30% acetonitrile for the next sample.

### 2.3. Gel-Filtration and Enzyme Inhibition Kinetics Analysis of Curcumin-Treated PMPMEase

To determine the inhibition mechanism of PMPMEase by curcumin, PMPMEase (1 mg) was preincubated with or without curcumin (10 *μ*M) for 60 min in identical conditions as in the enzyme assays except that no substrate was included. These were then fractionated on a Superdex 200 gel-filtration column (2 cm ID × 90 cm), eluting with 50 mM Tris-HCl (pH 7.4) containing 0.1% Triton X-100 and 0.5 M NaCl. Aliquots of the 4 mL fractions were then analyzed for enzyme activity using RD-PNB as the substrate. 

Michaelis-Menten kinetics analysis was conducted using RD-PNB as the substrate as previously described [38]. Varying concentrations of the substrate (0–400 *μ*M) were incubated with PMPMEase (5 *μ*g) in the presence of curcumin (0-1 mM). The reactions were carried out in 100 mM Tris-HCl, pH 7.4 containing 5% DMSO at 37°C in a total incubation volume of 100 *μ*L. Reactions were stopped by adding 200 *μ*L of methanol. They were then placed on ice for at least 5 min before centrifugation at 5000 ×g for 5 min. The supernatants were removed and analyzed by RP-HPLC with UV detection at 260 nm as previously described [38]. The product peak areas were measured and used to quantify the amount of product formed using a calibration plot of known amounts of product against peak area. All experiments were conducted in triplicates.

### 2.4. Docking Analysis

Docking was employed to determine the putative binding interactions of curcumin to PMPMEase. PMPMEase shares 79% sequence identity and 88% sequence similarity to human carboxylesterase 1 (hCE1) [36]. As previously described, the X-ray crystal structure of hCE1 [EC 3.1.1.1], 1YAH was used to construct the porcine liver esterase (PLE) structure for docking analysis [38]. Docking analysis was performed with Tripos SYBYL-X (v. 1.3). DScore was used for the evaluation of PMPMEase (1YAH) and PLE. Docking was carried out according to the developer's instructions [54]. In a crystallized receptor-ligand complex, the ligand was extracted from the enzyme, and the binding sites over the whole protein were detected using a multiresidue search. SYBYL-X discovered 10 possible binding sites on 1YAH and 9 sites on PLE. A total of 30 poses were observed and scored for each model and each binding site. The top ranking docking poses with minimal binding free energies were examined and noted in Table 1.

### 2.5. Cell Culture Conditions and Viability Assays

Caco-2 cells were cultured to 80–90% confluence, trypsinized and seeded onto 96-well plates at a density of 2.5 × 10^4^ cells/well and incubated for 24 h at 37°C in 5% CO_2_/95% humidified air. The cells were then exposed to varying concentrations of curcumin (0–200 *μ*M) in serum-free media daily for 72 h. Resazurin (Promega, WI, USA) was used to measure the cell viability according to the vendor instructions. Resazurin (20 *μ*L) was added to each well, and the contents were gently mixed and incubated in the dark for 2 h at room temperature before measurement of the fluorescence with excitation at 560 nm and emission at 590 nm using FLx 800 Microplate Fluorescence Reader (Bio-Tek Instruments Inc., VM, USA). Cell viability was expressed as the percentage of the fluorescence in the treated cells relative to that of the controls. The data are mean values from three different experiments.

### 2.6. Determination of PMPMEase Activity in Curcumin-Treated Cells

Cells were cultured to 80–85% confluence. Trypsin-EDTA (0.25%) was used to detach the cells. The cells were thoroughly washed with PBS and lysed with 0.1% Triton-X 100 in 100 mM Tris-HCI buffer, pH 7.4. Aliquots of the resulting lysate were preincubated for 15 min with curcumin (0–1000 *μ*M) before the addition of substrate. RD-PNB (1 mM) was incubated at 37°C with the lysate in the presence of curcumin in 100 mM Tris-HCl, pH 7.4 in a total incubation volume of 100 *μ*L. Reactions were stopped by adding 200 *μ*L of methanol and placing them on ice for at least 5 min before centrifugation at 5000 ×g for 5 min. The supernatant was analyzed by RP-HPLC as described earlier under enzyme assays.

### 2.7. Tissue Microarray and Immunohistochemical Studies

The expression of PMPMEase in colon cancer tissues was studied using immunohistochemical analysis on a colon cancer, normal adjacent, and normal tissue microarrays (TMAs) composed of a total of 208 cores from 208 cases. The human TMAs used in the studies were supplied by, and the immunohistochemistry conducted at US Biomax (Rockville, MD). All the tissues were formalin-fixed, paraffin-embedded, and mounted on positively charged SuperFrost Plus glass slides. Tissue sections (5 *μ*m thick and 1 mm in diameter) deparaffinized and hydrated were subjected to antigen retrieval in a microwave for 20 min in antigen retrieval solution (DAKO Corporation, CA, USA) and cooled for 15 min. As described in [55], the slides were incubated for 1 h with rabbit polyclonal antibody directed against PMPMEase (human carboxylesterase 1, hCE1) (Santa Cruz Biotechnology, CA, USA) diluted to a final concentration of 0.25 *μ*g/mL. Slides were then incubated with the secondary antibody using the ImmPRESS Reagent anti-Rabbit IgG (Vector Laboratories, CA, USA). Staining was performed with 3,3′-diaminobenzidine (DAB) as a chromogen, and sections were then counterstained with hematoxylin QS (Vector Laboratories, CA, USA). The IHC-stained slides were scanned at 20x magnification.

The method used to score the PMPMEase immunoreactivity was adapted from that of Bremnes et al. [56]. The intensity of the staining was given scores of 0 (no staining), 1 (trace), 2 (weak), 3 (intermediate), 4 (strong), and 5 (very strong). The score of the staining intensity was then multiplied by the percentage of the immunoreactive tumor cells. The overall scores ranged between 0 and 500 with those between 0 and 100 described as trace, 101 to 200 as weak, 201 to 300 as intermediate, 301 to 400 as strong, and 401 to 500 as very strong. The evaluation and scoring were conducted by RD, FA, BJA, and RP without prior knowledge of the diagnosis of the individual cores on the TMAs.

### 2.8. Oncomine Cancer Microarray Database Analysis

The Oncomine Cancer Microarray database (http://www.oncomine.org/) was used to study the profile of PMPMEase gene expression in human colorectal cancer. “Colorectal cancer and CES1” as well as “colon cancer and CES1” were typed into the search window. All studies involving colorectal cancer with CES1 expression profiles were considered for study. The gene expression data from each study, performed with the same methodology, were used. The gene expression data were log transformed, and a gene was considered as overexpressed when the fold change in the level of expression was ≥1.0. 

### 2.9. Statistical Analysis

All results were expressed as the means ± S.E.M. The concentration-response curves were obtained by plotting the percentage residual PMPMEase activities against the log of curcumin concentrations. Nonlinear regression plots were generated using Graphpad Prism version 4.0 for Windows (San Diego, CA, USA). From these, the concentrations that inhibit 50% of the activity (IC_50_) were calculated. The TMA data were analyzed by one-way ANOVA using SAS 9.2 software (SAS Institute, NC, USA) followed by Bonferroni's procedure for multiple comparisons [57]. *P*-values of less than 0.05 were considered statistically significant. 

## 3. Results 

### 3.1. PMPMEase Activity Is Inhibited by Curcumin

**Figure 1:**
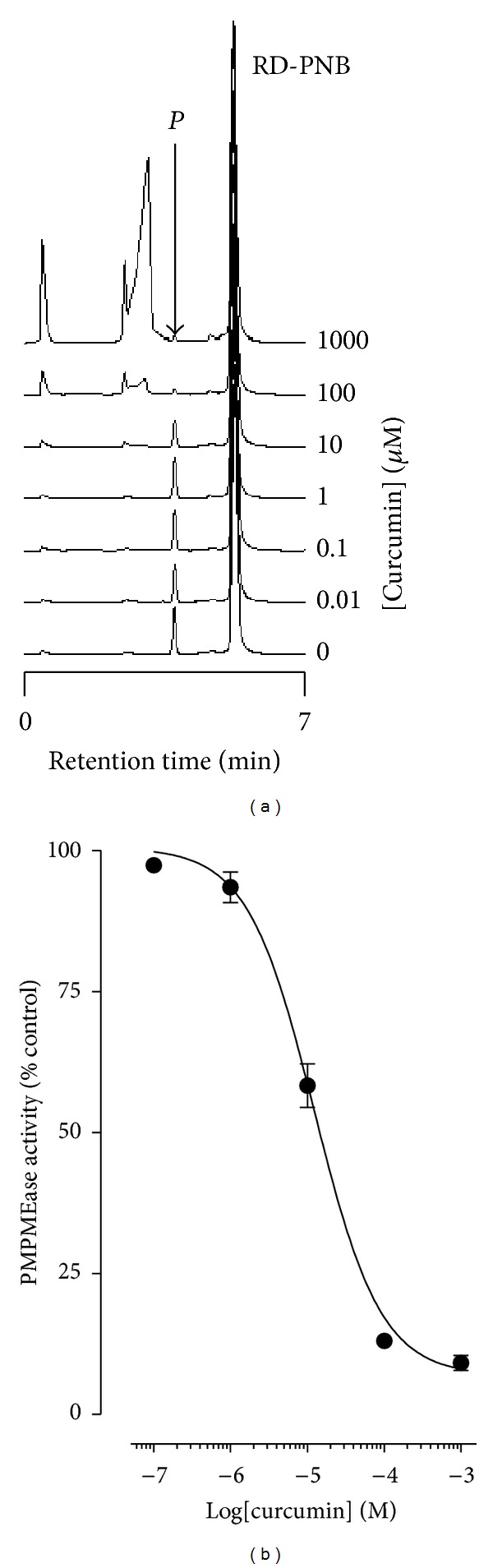
Inhibition of PMPMEase by curcumin. (a) Purified PMPMEase (5 *μ*g) was incubated with RD-PNB in the presence of varying concentrations of the indicated concentrations of curcumin for 1 h. The reactions were stopped with methanol and analyzed for the residual PMPMEase activity as described in the methods section. (b) The results are expressed as the means relative to the controls (±SEM, *N* = 3).

When the RD-PNB substrate was incubated with purified PMPMEase in the presence of curcumin, the hydrolysis of the substrate was inhibited as denoted by significant decreases in product formation. As shown in Figure 1(a), maximum enzymatic activity was achieved in the absence of curcumin indicated by large product peak area. With increasing concentration of curcumin, there was a progressive decrease in the product peak area. When the relative amounts of product formed were plotted against the respective curcumin concentrations (Figure 1(b)), an IC_50_ value of 12.4 *μ*M (4.4 *μ*g/mL) and corresponding *K*_*i*_ of 0.31 *μ*M were obtained. 

### 3.2. Curcumin Inhibition of PMPMEase Is Reversible

**Figure 2:**
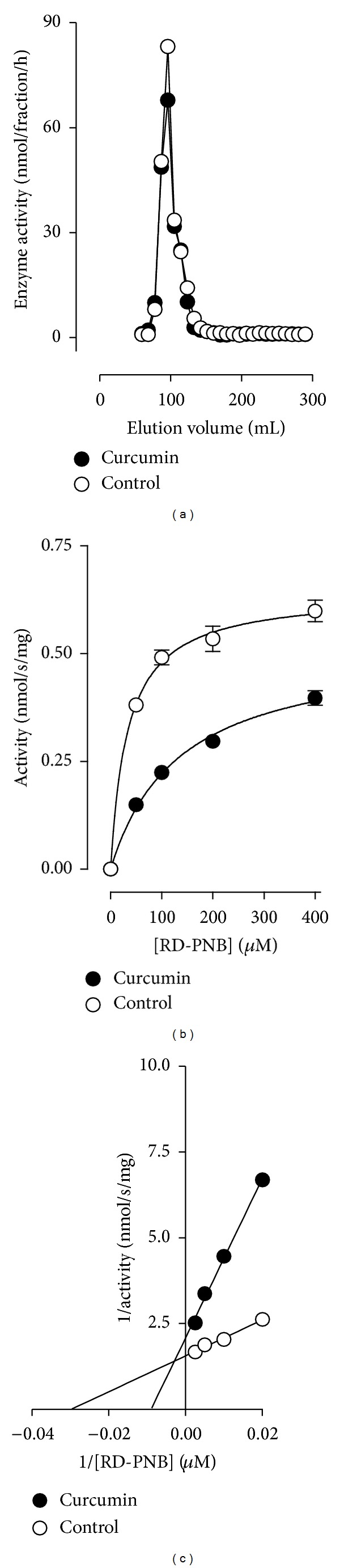
Gel-filtration analysis of curcumin-treated PMPMEase. PMPMEase was preincubated with 10 *μ*M curcumin followed by gel-filtration chromatography to separate free curcumin from the enzyme. Aliquots of the collected fractions were assayed for residual PMPMEase activity (a). Michaelis-Menten kinetics (b) and double reciprocal analyses of the inhibition of PMPMEase by curcumin (c). Curcumin treatment (closed circles ●) was compared to untreated control (open circles ○).

Preincubation of PMPMEase with 10 *μ*M curcumin followed by gel-filtration chromatography resulted in the recovery of virtually all of the enzyme activities. The comparable activities detected in analogous fractions of the curcumin-treated and untreated PMPMEase samples following gel-filtration chromatography (Figure 2(a)) are indicative of a reversible inhibition mechanism. Michaelis-Menten kinetics analysis with the RD-PNB substrate also revealed a possible mixed inhibition mechanism for curcumin against PMPMEase as depicted by changes in both Michaelis-Menten constants and *V*_max⁡_ values in the presence of 20 *μ*M curcumin (Figures 2(b) and 2(c)). The *K*_*M*_ for RD-PNB metabolism by PMPMEase was 23.6 ± 2.7 and 85.3 ± 15.3 *μ*M in the absence or presence of 20 *μ*M curcumin, respectively. On the other hand, over 3-fold change in *K*_*M*_ was associated with a lesser change in the *V*_max⁡_ from 0.60 ± 0.02 to 0.45 ± 0.03 nmol/s/mg for the uninhibited and inhibited reactions, respectively.

### 3.3. Molecular Docking Analysis Reveals Multiple Curcumin Binding Sites on PMPMEase

**Figure 3:**
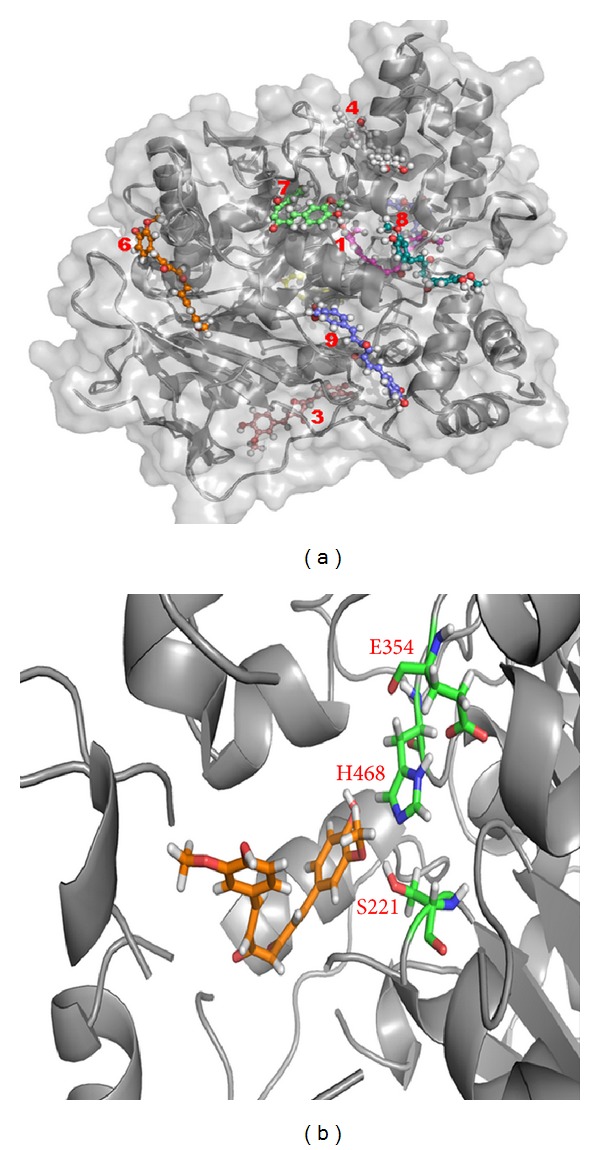
Docking analysis of curcumin binding to PMPMEase. (a) The crystal structure of hCE1/human PMPMEase (1YAH) enzyme showing the docking of curcumin at the active and allosteric sites displayed in the Licorice visualization. The visualizations were created using visual molecular dynamics (VMD). (b) Curcumin in the active site of 1YAH in the Licorice visualization. The active site catalytic triad of amino acids is shown with the coloring method (carbon atoms in blue, oxygen in red, nitrogen in dark blue, and hydrogen in white). Curcumin is shown in orange Licorice visualization.

While curcumin inhibits PMPMEase substantially, the mode of inhibition is unknown. Docking analysis of curcumin to PMPMEase revealed multiple binding sites that included one active site binding interaction and 9 allosteric sites (Figure 3). The DScore binding affinities of curcumin and the corresponding binding sites are shown in Table 1. The binding affinities measured in kcal/mole ranged from −109.73 to −206.74 for the respective binding sites of 1YAH. The specific binding energy at the active site (site 1) was −187.40 kcal/mole. This is consistent with the Michaelis-Menten kinetics analysis results (Figure 3). 

### 3.4. PMPMEase Inhibition by Curcumin Reduces Colorectal Cancer Cell Viability

**Figure 4:**
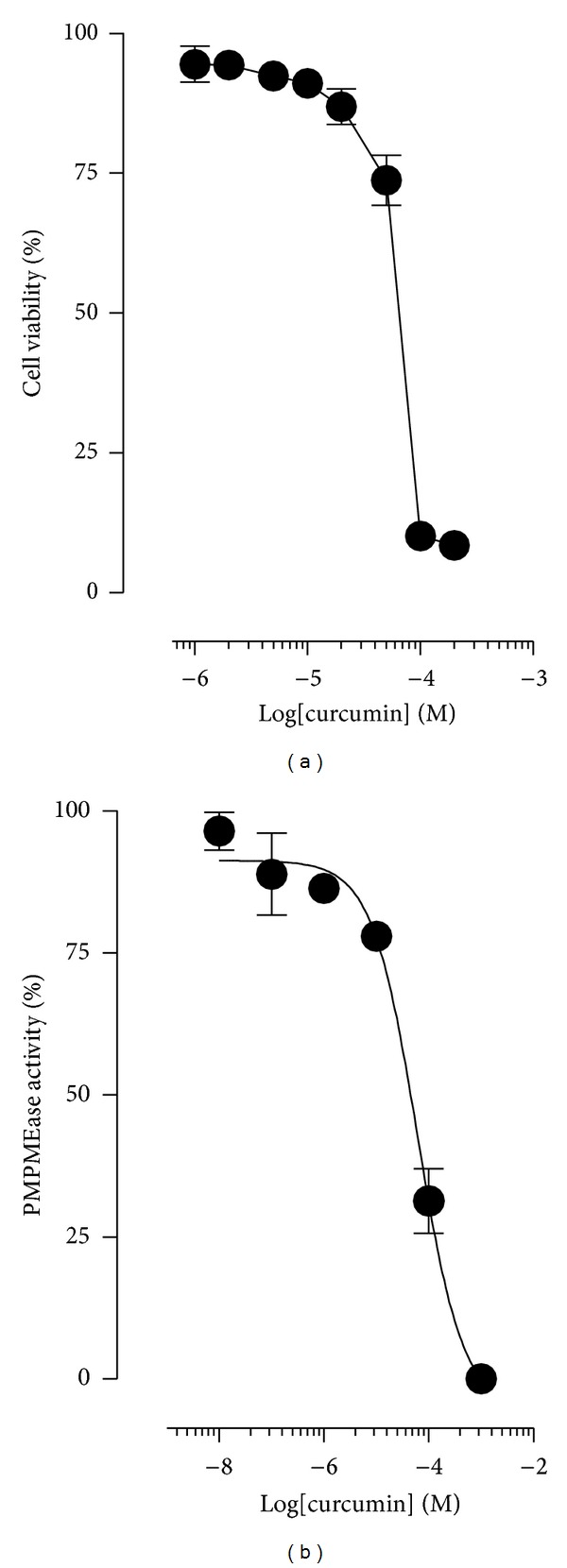
(a) Curcumin induced degeneration of human colorectal cancer Caco-2 cells. Human colorectal cancer Caco-2 cells were cultured and seeded in 96-well plates at a density of 2 × 10^4^ as described in the methods. At 72 h after treatment with varying concentrations of curcumin, cell viability was measured by fluorescence using the resazurin reduction assay. Each data point represents the mean ± SEM of 4 wells. The data are representative of 3 separate experiments (EC_50_ = 22.0 *μ*g/mL). (b) PMPMEase activity in degenerating curcumin-treated human colorectal cancer Caco-2 cells. Cells were cultured to 80% confluence, lysed, and incubated with the indicated concentrations of curcumin as described in the methods. The residual PMPMEase activity was then determined using RD-PNB as the substrate. Each point represents the mean ± SEM (*n* = 3). The data are representative of 3 separate experiments (IC_50_ = 22.6 *μ*g/mL).

Malfunctions of polyisoprenylated proteins contribute to aberrant signaling resulting in the progression of several cancers. For example, Ras mutations that occur in over 50% of colorectal cancer cases are associated with a more aggressive disease [58]. Compounds that inhibit PMPMEase may contribute to the prevention or treatment of colorectal cancer. Cytotoxicity studies with Caco-2 cells using curcumin have yielded interesting results, and several mechanisms have been proposed for the apoptotic effects [30, 59–61]. In a study using curcumin and celecoxib, Lev-Ari and coworkers [59] proposed a synergistic inhibition of the COX-2 pathway for the inhibition of cell growth in HT-29 and IEC18-K-ras cells that express high levels of COX-2. However, no plausible conclusion could account for similar additive growth inhibition seen in colorectal cancer cell lines that expressed low or no COX-2 activity (Caco-2 and SW-480) [59]. We therefore sought to understand if the inhibitory effect of curcumin on PMPMEase could account for its anticancer effects using Caco-2 cells. Treatment of Caco-2 cells with curcumin resulted in the concentration-dependent inhibition of both cell viability (EC_50_ of 60 *μ*M or 22.0 *μ*g/mL) and cellular PMPMEase activity (IC_50_ of 61 *μ*M or 23 *μ*g/mL) (Figure 4). The loss of cell viability due to PMPMEase inhibition has been demonstrated in a wide variety of cell lines in our laboratory, including human neuroblastoma (SH-SY5Y) cells, human lung cancer (A549 and H460) cells, human triple negative breast cancer MDA-MB-231 cells, human pancreatic (BxPC-3) cells, and human prostate cancer (LNCaP) cells [33–35, 62]. Moreover, the EC_50_ for cell viability of 22 *μ*g/mL obtained in this study is less than the mean curcumin level of 48.4 *μ*g/g detected in human colorectal tissue biopsies after daily oral doses of 2.35 g curcuminoids [63], bearing in mind a tissue density of about 1.06 g/mL. 

### 3.5. PMPMEase Is Overexpressed in Colon Cancer

**Figure 5:**
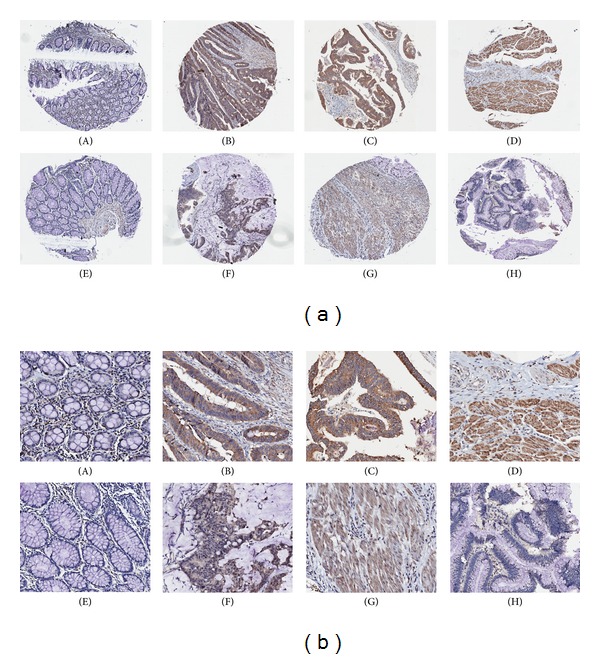
(a) Immunohistochemical analysis of TMA cores from colon cancer cases showing brown staining for PMPMEase immunoreactivity. The TMAs were probed with PMPMEase antibodies and scored for the relative intensities of PMPMEase staining as described in the methods. Intense staining was observed in colon adenocarcinoma (stage I, B, C and D), mucinous adenocarcinomas (stage 2, F), signet ring cell carcinoma (stage II, G) and papillary adenoma (stage 2, H). A and E are images of sections obtained from normal colon tissue and normal adjacent tissue, respectively. Each image is of a section from the tumor of a separate case. (b) Magnified sections of colon adenocarcinoma (B, C and D), mucinous adenocarcinomas (F), signet ring cell carcinoma (G) and papillary adenoma (H). Areas with dense populations of blue-stained nuclei indicative of tumor cells also show a higher intensity of brown staining for PMPMEase. A and E are magnified images of sections obtained from normal colon and normal adjacent tissues, respectively.

The colon cancer TMA was analyzed for the relative expression of PMPMEase. The demographic and histopathological characteristics of the tissue donors for the TMAs are shown in Table 2. The ages of the patients ranged from 23 to 90 years, and most of them (67.3%) were males. There were 175 cases of adenocarcinoma, 15 cases of mucinous adenocarcinoma and 1 case each of papillary adenoma and signet ring cell carcinoma. In general, 88.6% of the colon cancers showed intracellular PMPMEase immunoreactivity. The data indicate that increasing levels of PMPMEase expression are associated with tumors (Figure 5(a)). In the control cores consisting of normal colon tissues and normal adjacent colon tissues, either negative or focal mild cytoplasmic immunostaining was observed. Meanwhile, solid tumors with intensely stained cells were displayed especially in most of the colon adenocarcinoma, mucinous adenocarcinomas, and signet ring cell carcinoma. Figures 5(a) and 5(b) show representative images of normal colon and colon cancer tissues. Significant differences in PMPMEase immunoreactivity intensities between the normal tissues and the different colon tumor categories were observed when the IHC-stained sections were analyzed (*P* = 0.0002–<0.0001) (Table 3). Paired comparisons of immunoreactivity scores for PMPMEase proteins between normal tissues versus tumors and normal adjacent tissues versus metastatic tumors were significant (*P* < 0.0001). The mean scores ± SEM were 91.7 ± 11.4 for normal, 75.0 ± 14.4 for normal adjacent, 294.8 ± 7.8 for adenocarcinoma, and 310.0 ± 22.6 for mucinous adenocarcinoma. Relatively high PMPMEase expression (score = 301–400) was observed in both the papillary adenoma and signet ring cell carcinoma. Although no specific trend was observed when the data were analyzed according to pathological stages, grades, tumor size, nodal status, and metastases, there were significant differences when compared to the normal colon tissues and the NATs regardless of the parameter under consideration. Taken together, these findings show that PMPMEase protein is overexpressed in colorectal cancer. 

### 3.6. PMPMEase Gene Is Overexpressed in Colon Cancer

The Oncomine database queried to systematically assess relative gene expression levels of PMPMEase (CES1) genes in colorectal tumors. We have analyzed the studies that showed a significant fold change gene expression (*P* ≤ 0.001) in cancerous tissues. Although some of the studies showed some cases of downregulation of PMPMEase [40, 45, 47], most of the studies retrieved showed significant overexpression of the PMPMEase (Table 4).

## 4. Discussion

Despite the remarkable recent advances in surgical excision, radiotherapy, and chemotherapeutic regimens, the high recurrence rates and fatalities from colorectal cancer [64] imply that its management remains an area of unmet medical need. Prevention plays a vital role in limiting the impact of the disease. Understanding the mechanisms by which bioactive substances such as curcumin exert their pharmacological effects is essential for maximizing the health benefits. Therefore, identifying the target with which curcumin interacts is essential for fully understanding its mechanism of action. In this study, we determined that PMPMEase, while being overexpressed in colon cancer, is also susceptible to inhibition by curcumin. These interesting observations are pertinent in two respects (i) that PMPMEase, given previous studies linking its inhibition to cancer cell death [33–35], likely contributes to at least some cases of colorectal cancer progression and (ii) that the widely reported chemopreventive effects of curcumin [18, 19, 26] are due at least in part to PMPMEase inhibition. The elevation of PMPMEase protein levels in this study is corroborated by previous studies in which mRNA levels were determined to be significantly higher in some cases of colorectal cancers as revealed by the Oncomine database analysis. 

The overexpression of PMPMEase in colon cancer is significant in view of the role that polyisoprenylated proteins play in cell growth and motility. Mutant constitutively active forms of members of the Ras superfamily of proteins are observed in about 50% of colorectal cancer cases [32, 58, 65]. More importantly, signaling pathways involving aberrant activities of these monomeric G-proteins are common in colorectal cancer [11, 32]. Gulhati and coworkers [11] reported that mTORC1 and mTORC2 regulate changes in the actin cytoskeleton and cell migration by signaling through RhoA and Rac1 pathways [11]. The Rho family of GTPases are involved in the formation of lamellipodia and cell migration [10]. Also, while mutations and/or overexpression are linked to their tumorigenic activities, secondary modifications are essential for their normal and pathological activities [32, 66]. Although the polyisoprenylation pathway enzymes have been the subjects of pharmaceutical development efforts [12], the role of PMPMEase in regulating polyisoprenylated protein function is only just getting attention [67]. Therefore the overexpression of PMPMEase in colon cancer highlights its role as a potential target for curcumin and other food-derived bioactive compounds. Its putative endogenous substrates include not only the monomeric G-proteins but also the heterotrimeric G-protein-coupled receptors (GPCRs) whose contributions in cancers have also been widely reported [32, 66]. GPCRs constitute a large family of plasma membrane receptors that rely on the heterotrimeric G-proteins for intracellular signal transduction [68]. The *γ*-subunits of these trimeric complexes are polyisoprenylated, a feature that is essential for their functions [69]. Signaling through GPCRs such as some eicosanoid [70, 71], chemokine [72, 73], and adrenergic [74, 75] receptors play important roles in human colorectal cancer growth and metastasis. A recent study revealed that galanin receptor 1 (GalR1) and its ligand galanin are key determinants of drug resistance and potential therapeutic targets for combating drug resistance [76]. Bearing in mind that PMPMEase is one of two enzymes that catalyze reactions in the only reversible step of the pathway, its hyperactivity is bound to distort the equilibrium in favor of cell growth stimulation. 

The observation that curcumin inhibits PMPMEase is pertinent for our understanding of the chemopreventive mechanism of this important food-derived agent [77]. Although several mechanisms of action have been proposed for curcumin [27, 30, 31, 77], they do not appear to exclude the involvement of PMPMEase as an intermediary since effects on polyisoprenylated protein metabolism inevitably impact transcriptional activity. For example, curcumin has been shown to downregulate the expression of EGFR, COX-2, LOX, NOS, MMP-9, uPA, TNF, chemokines, cell surface adhesion molecules, cyclin D1, the transcription factors NF-*κ*B, AP-1, Egr-1 as well as inhibiting c-Jun N-terminal, protein tyrosine, and protein serine/threonine phosphorylation [30, 31, 77]. We previously demonstrated that PMPMEase is inhibited by PUFAs but not by prostaglandins [35]. The overexpression of PMPMEase in colorectal cancer, its inhibition by curcumin and its differential susceptibility to the PUFAs and PGs are significant against the backdrop of COX-2 overexpression especially in colorectal cancer. Furthermore, long-term use of NSAIDs is associated with lower cancer risks [78, 79]. Considering this and the numerous reports that COX-2 and PGs are important in the development and progression of cancers [80], it has been opined that COX-2-selective inhibition holds a promising role in cancer chemoprevention [78]. Therefore a mechanism for curcumin action that involves the suppression of PUFAs-oxidizing enzymes would be consistent with preserving the PUFAs for PMPMEase inhibition. An inhibited PMPMEase is likely to modulate the actions of polyisoprenylated proteins such as Ras and its signaling pathways as previously reported [31]. Curcumin and its derivatives have also been reported to inhibit farnesyl transferase, a polyisoprenylation pathway enzyme essential for the transformation of Ras into its biologically active form [81, 82]. That curcumin's anticancer activities are mediated through PMPMEase inhibition is further substantiated by our previous findings in which PMPMEase inhibition with specifically designed polyisoprenylated sulfonyl fluorides resulted in cancer cell death [33]. That such a profound cellular effect occurs upon PMPMEase inhibition has been explained by the significant conformational changes near the physiologically important polyisoprenyl moiety of the signaling proteins. The polyisoprenyl moiety is pertinent for the functional interactions of polyisoprenylated proteins with other proteins [83]. The charge difference due to the change in methylation-demethylation balance is believed to be similar in effects on conformations as do the phosphorylation-dephosphorylation of kinase-regulated proteins [84]. 

The observation through docking analysis that curcumin may interact competitively with the active site and allosterically with other sites likely explains the mixed antagonism characteristics observed with the Michealis-Menten kinetics analysis. The susceptibility of PMPMEase to PUFAs and curcumin suggests that it may be a target for other food-derived anticancer agents. Food-derived agents, especially flavonoids such as *all trans* geranylgeraniol, farnesol-mixed isomers, *trans trans *farnesol, eugenol, *α*-Ionone, and 2,3-heptanedione have structures that resemble the polyisoprenes. These hydrophobic molecules are of the appropriate sizes to enter the active site and competitively inhibit the enzyme. The active site of PMPMEase is large, flexible, and lined with hydrophobic aromatic amino acid residues [85]. This property promotes binding to a wide variety of mainly hydrophobic molecules [85] while also precluding oxidized more hydrophilic analogs [35]. 

The current findings further reveal the pertinent role that PMPMEase plays in colorectal cancer and how its levels of activity and expression can be exploited in companion diagnosis. It is also increasingly apparent that PMPMEase is susceptible to inhibition by various food-derived chemopreventive agents thus implying that a systematic screening of such substances may reveal a repertoire of such compounds for nutraceuticals. Several studies using various animal models or human subjects indicate that curcumin is very safe, even at a very high dose of 12 g per day. This dose can easily be obtained when curcumin is included in food as a spice and/or a food preservative. Howells et al. [86] showed that low doses of curcumin produced colonic tissue concentrations of an order of magnitude associated with pharmacological effects, both in cells *in vitro* and in rodents *in vivo*. Ravindranath and Chandrasekhara [87] showed that, after oral administration of curcumin (2 g/kg), the levels detected in the stomach, small intestine, cecum, and large intestine were 53.3 ± 5.1 *μ*g/g, 58.6  ±  11.0 *μ*g/g, 51.5 ± 13.5 *μ*g/g, and 5.1 ± 2.5 *μ*g/g, respectively. In a more recent study by Irving et al. [63], daily oral curcuminoids (2.35 g) resulted in a mean curcumin level colorectal tissue biopsies of 48.4 *μ*g/g without prior washing which has been a standard practice for both clinical and *in vivo* studies. However, washing the tissue reduced this difference to only 2-fold with mean washed tissue levels of 18.85 *μ*g/g. Therefore, the EC_50_ for cell viability of 60 *μ*M (22 *μ*g/mL) obtained in this study is within the range achievable *in vivo,* assuming average tissue densities of about 1.06 g/mL. 

Finally, these studies strongly suggest that potent, rationally designed PMPMEase inhibitors would be invaluable therapeutic agents in the management of those colorectal cancers cases in which PMPMEase expression and activities are elevated. 

## 5. Conclusion 

In summary, elevated PMPMEase activity and its overexpression in colon cancer makes it a suitable biomarker that can be developed into a procedure for the early/companion diagnosis of colon cancer. The susceptibility of PMPMEase to PUFAs and curcumin suggests that it may be a target for other food-derived anticancer agents. Potent and specific inhibitors of PMPMEase could eventually be developed as a new class of targeted therapies for colorectal cancers cases in which PMPMEase expression and activities are elevated.

## Figures and Tables

**Table 1:** Docking data for the binding affinities of curcumin with the respective binding sites.

Docking site	Tripos SYBYL-X Scoring Functions	
	DScore (Kcal/mol)	
	1YAH	PLE
1	−187.40	−944.63
2	−206.74	−581.16
3	−118.87	−1089.5
4	−155.45	−930.83
5	−109.73	−790.88
6	−114.69	−733.22
7	−183.87	−624.60
8	−114.84	−690.98
9	−114.11	−523.63
10	−170.18	N/A

**Table 2:** Demographic, histopathological characteristics and the disease states of the 208 donors of the colon tissues used in the tissue microarray studies.

Characteristics	Patients	
	*n*	(%)
Age		
≤65 years	152	73.1
>65 years	56	26.9
Sex		
Female	68	32.7
Male	140	67.3
Histology		
Normal	12	5.8
NAT	4	1.9
*Adenocarcinomas *	175	84.1
*Mucinous adenocarcinoma *	15	7.2
*Papillary adenoma *	1	0.4
*Signet ring cell carcinoma *	1	0.4
Grade		
1	35	16.8
2	101	48.6
2-3	10	4.8
3	22	10.6
Not determined	24	11.5
Pathological stage		
I	92	44.2
II	89	42.8
III	6	2.9
IV	5	2.4
Tumor status		
1	1	0.4
2	53	25.5
3	90	43.3
4	48	23.1
Nodal status		
0	176	84.6
1	13	6.3
2	1	0.4
Metastasis		
0	187	89.9
1	4	1.9

**Table 3:** Association of PMPMEase immunoreactivity with the pathologic features of colon cancer. Significantly higher PMPMEase immunoreactivities were observed in the different types, grades, and stages of colon cancers as shown by the means ± SEM versus normal tissues compared by ANOVA followed by Dunnet's post test.

Characteristics	PMPMEase Staining Intensity, *N* (%)					Missing	Mean Scores	*P*-value
	1–100 Trace	101–200 Weak	201–300 Intermediate	301–400 Strong	401–500 Very strong			
Normal	11 (75.0)	3 (25.0)	0	0	0	0	91.7 ± 11.4	
NAT	4 (100)	0	0	0	0	0	75.0 ± 14.4	
Histology								
*Adenocarcinomas *	7 (4.0)	30 (17.1)	57 (32.6)	54 (30.9)	20 (11.4)	7 (4.0)	294.8 ± 7.8	0.0001
*Mucinous adenocarcinoma *	1 (6.7)	1 (6.7)	6 (40.0)	6 (40.0)	1 (6.7)	0	310.0 ± 22.6	
*Papillary adenoma *	0	0	0	1 (100.0)	0	0		
*Signet ring cell carcinoma *	0	0	0	1 (100.0)	0	0		
Pathological stage								
I	2 (2.2)	13 (14.1)	33 (35.9)	32 (34.8)	10 (10.9)	2 (2.2)	306.1 ± 10.0	0.0001
II	5 (5.6)	16 (18.0)	26 (29.2)	28 (31.5)	11 (12.4)	3 (3.4)	291.6 ± 11.2	
III	1 (16.7)	1 (16.7)	1 (16.7)	2 (33.3)	0	1 (16.7)	270.0 ± 53.3	
IV	0	1 (20.0)	3 (60.0)	0	0	1 (20.0)	218.8 ± 35.9	
Grade								
1	2 (5.7)	5 (14.3)	8 (22.9)	18 (51.4)	2 (5.7)	0	306.4 ± 17.1	0.0001
2	4 (4.0)	22 (21.8)	37 (36.7)	25 (24.8)	10 (9.9)	3 (3.0)	281.6 ± 9.9	
2-3	0	0	5 (50.0)	5 (50.0)	0	0	302.5 ± 18.1	
3	2 (9.1)	3 (13.6)	10 (45.5)	5 (22.7)	2 (9.1)	0	270.5 ± 21.7	
Not determined	0	1 (4.2)	3 (12.5)	9 (37.5)	7 (29.2)	4 (16.7)	377.5 ± 18.6	

**Table 4:** PMPMEase (CES1) gene is overexpressed in human colorectal cancers. Oncomine studies used in this analysis are shown below.

Cases	Number of cases with fold change greater than 2		Year of study	References
	Downregulated	Upregulated		
70	7 (10%)	28 (40%)	2010	[39]
22	5 (22%)	12 (55%)	2007	[40]
48	1 (2%)	16 (33%)	2006	[41]
100		33 (33%)	2007	[42]
12		7 (58%)	2001	[43]
13		9 (69%)	2003	[44]
154	10 (7%)	50 (32%)	2009	[45]
23		3 (13%)	2008	[46]
80	12 (15%)	58 (73%)	2007	[47]
55	2 (4%)	9 (16%)	2007	[48]
177		59 (33%)	2010	[49]
62		7 (11%)	2009	[50]
42	4 (10%)	14 (33%)	2006	[51]
104		18 (17%)	2011	[52]
176		82 (46%)	2011	[53]

Number of cases with significant fold change (*P* < 0.001) and the percentage (in brackets) of the number of their respective cases are indicated.
